# Morphological Analysis of PSMA/PEI Core–Shell Nanoparticles Synthesized by Soap-Free Emulsion Polymerization

**DOI:** 10.3390/nano11081958

**Published:** 2021-07-29

**Authors:** Jae-Jung Park, Yongsoo Kim, Chanmin Lee, Donghyun Kim, Wonjun Choi, Hyukjun Kwon, Jung-Hyun Kim, Ki-Seob Hwang, Jun-Young Lee

**Affiliations:** 1 Department of Chemical and Biomolecular Engineering, Yonsei University, Yonsei-ro 50, Seodaemun-gu, Seoul 120749, Korea; qkrwownd11@yonsei.ac.kr (J.-J.P.); ratian36@kitech.re.kr (D.K.); qwerty042@kitech.re.kr (W.C.); hyukjun@kitech.re.kr (H.K.); jaykim@yonsei.ac.kr (J.-H.K.); 2 Research Institute of Sustainable Manufacturing System, Intelligent Sustainable Materials R&D Group, Korea Institute of Industrial Technology, 89 Yangdaegiro-gil, Ipjang-myeon, Seobuk-gu, Cheonan-si 31056, Korea; bohemian4215@kitech.re.kr (Y.K.); clee@kitech.re.kr (C.L.)

**Keywords:** core–shell nanoparticle, particle size control, poly(styrene-*co*-maleic anhydride), particle size distribution, morphology

## Abstract

Emulsion polymerization presents the disadvantage that the physical properties of polymer particles are altered by surfactant adsorption. Therefore, in the soap-free emulsion polymerization method, a hydrophilic initiator is utilized while inducing repulsion among particles on the polymer particle surface, resulting in stable polymer particle production. In this study, we developed a methodology wherein spherical and uniform poly(styrene-*co*-maleic anhydride) (PSMA)/polyethyleneimine (PEI) core–shell nanoparticles were prepared. Further, their morphology was analyzed. During PSMA polymerization, the addition of up to 30% maleic anhydride (MA) resulted in stable polymerization. In PSMA/PEI nanoparticle fabrication, the number of reactants increased with increased initial monomer feed amounts; consequently, the particle size increased, and as the complete monomer consumption time increased, the particle distribution widened. The styrene (St) copolymer acted as a stabilizer, reducing particle size and narrowing particle distribution. Furthermore, the monomers were more rapidly consumed at high initiator concentrations, irrespective of the initiator used, resulting in increased particle stability and narrowed particle distribution. The shell thickness and particle size were PEI feed ratio dependent, with 0.08 being the optimal PEI-to-MA ratio. The fabricated nanoparticles possess immense potential for application in environmental science and in chemical and health care industries.

## 1. Introduction

The synthesis of nanoparticles is a complex process; single nanoparticles were the focus of research in the early days, but since the late 1980s, studies have found that hybrid or complex colloid particles have a higher efficiency. To enhance the properties of these materials, multilayered nanoparticles have been synthesized since the early 1990s, during which the term “core–shell” was introduced. Unlike single nanoparticles, composite nanoparticles and core–shell particles are composed of at least two materials [1,2,3,4,5,6]. Core–shell nanoparticles consist of a core (internal material) and a shell (external material), which leads to a broad definition; based on the purpose of use, the choice of material can be varied among the inorganic–inorganic, inorganic–organic, organic–inorganic, and organic–organic composites [7,8,9,10]. Core–shell nanoparticles have applications in various fields such as materials chemistry, electronics, engineering, biomedicine, pharmaceuticals, optical science, and catalysis [11,12,13,14]. The characteristics of core–shell nanoparticles can be regulated by modifying the components and the composition ratio of the core to shell materials [15,16,17,18].

A copolymer of styrene (St) and maleic anhydride (MA), known as polystyrene-*co*-maleic anhydride (PSMA), and polyethylene imine (PEI) were used as the core and shell, respectively, to fabricate core–shell nanoparticles. The core-PSMA is used as a drug carrier, chromatography column packaging material, device calibration reference, and template for the preparation of porous materials [19,20]. The shell-PEI is used in biomedical science owing to its nontoxicity [21]. Various methods have been suggested for the polymerization of core-PSMA, namely suspension polymerization, emulsion polymerization, dispersion polymerization, and soap-free emulsion polymerization; among these, emulsion polymerization is useful for obtaining particles of sizes 50–600 nm [22,23,24,25,26].

Small-molecule surfactants are used in traditional emulsions to reduce the interfacial tension between two immiscible liquids and maintain the emulsion stability. However, molecular surfactants are highly toxic, polluting, and difficult to recover [27].

Emulsion polymerization presents a problem where the physical properties of polymer particles are altered when surfactants adsorb to the polymer particle surface; the surfactants have a role in stabilizing the particle dispersion and forming the venue of reaction upon polymerization [28,29,30,31]. In addition, while removing the surfactants for using them, the polymer particles may be destabilized, resulting in agglomeration, and the surfactants may cause environmental pollution, thereby increasing the production cost. To overcome these challenges, a soap-free emulsion polymerization method was employed, wherein an initiator functioning as an emulsifier on the polymer particle surface is used to ensure the stability of polymer particles in the dispersion when radicals are formed. Because a surfactant is not used, soap-free emulsion polymerization can minimize particle surface contamination caused by surfactant adsorption and prevent a decrease in dispersion stability upon removal of the emulsifier [32,33,34].

Ohtsuka et al. reported the preparation of surfactant-free PS-b-PAM. Initially, AM polymerizes in the aqueous phase and forms micelle-like PAM particles. The polymerization of styrene (St) occurs within hollow PAM particles, while AM continues to polymerize in the outer part of the PAM shell. The emulsions produced formed films, which were of good transparency in the absence of surfactant [35,36].

In the soap-free emulsion polymerization method, a hydrophilic initiator is utilized while inducing a repulsion among particles on the polymer particle surface, resulting in the production of stable polymer particles. Thus, the disadvantage of the potential alteration of the produced polymer particles upon removing the surfactants, as in emulsion polymerization, can be reduced. The rate of nucleation in soap-free emulsion polymerization is related to the solubility and dispersity of monomers, in addition to the rate of radical generation. A stable polymer emulsion is formed by the initiator, monomer, reaction temperature, and solvent. These characteristics have been exploited by Xing and Yang in their study, wherein alkyl esters were used as the reaction media, and a novel polymerization method was used to synthesize MA/VAc copolymerized nanoparticles; however, a detailed explanation of the effect of the surface reaction on particle formation was not provided in the report [37,38].

The synthesis technique and characteristics of core-PSMA nanoparticles are discussed in this article. An understanding of the mechanism of soap-free emulsion polymerization and the conditions for regulating the particle size and particle distribution were established. In addition, morphological analysis was performed. By examining the effect of the initiator type, concentration, total amount and ratio of monomers, polymerization time, and temperature that collectively determine the rate of polymerization, the optimum conditions were determined. The core–shell PSMA/PEI nanoparticles were fabricated using the established conditions. Furthermore, by estimating the appropriate amount of PEI, the optimum conditions for obtaining the desired particle size and distribution were ascertained for the first time, and a relevant morphological analysis was performed. Furthermore, the effects and mechanisms of these factors on the properties of the synthesized particles were investigated.

## 2. Materials and Methods

### 2.1. Materials

All chemical reagents used here were general-purpose reagents, and deionized (DI) water was used to prepare all the solutions. Styrene (99% purity) and the inhibitor removal column were purchased from Sigma-Aldrich, Inc. (St. Louis, MO, USA). Prior to polymerization, St was eliminated from the inhibitor (4-tert-butylcatechol) using an ammonium oxide column. Maleic anhydride (MA) and PEI (branched, average Mw = 25,000) were also purchased from the same company, and the initiators were from Daejung Chemicals and Metals Co. Ltd. (Siheung, Korea). Potassium persulfate (KPS) and 2, 2-Azobisisobutyronitrile (AIBN) were recrystallized in DI water and methanol, respectively.

### 2.2. Preparation of Core–Shell Nanoparticles

**Scheme 1:**
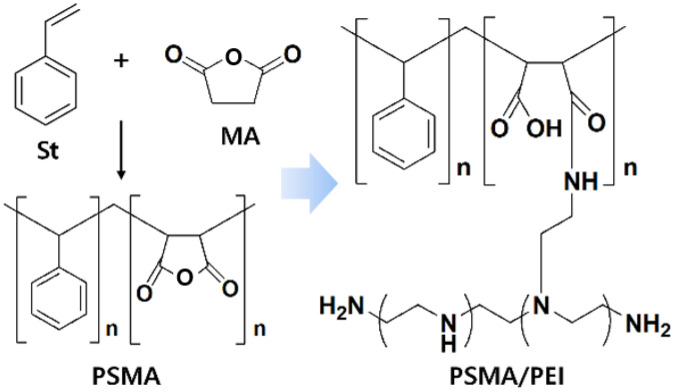
Schematic of the preparation of polystyrene-*co*-maleic anhydride/polyethylene imine (PSMA)/(PEI) core–shell nanoparticles.

The synthesis technique of a previous study [36] was used for fabricating the core-PSMA and PSMA/PEI core–shell structures (Scheme 1). A double-jacket reactor of 250 mL was equipped with a cooling condenser. The vacuum circulation pump (CW3-30) was activated in the reactor, and a nitrogen atmosphere was sustained by maintaining the temperature at 70 °C. Subsequently, PSMA was fabricated using soap-free emulsion polymerization with a mechanical stirrer (EUROSTAR 40 digital) rotating at 300 rpm.

Here, MA (0–30 wt%) was added to D.I water of 155 mL, which was placed in the reactor, and stirred for 10 min. Purified St (70–100 wt%) was added to the stirred MA and stirred for 30 min. To initiate the reaction, AIBN and KPS initiators were added after 30 min of stirring. The water-soluble KPS and lipid-soluble AIBN initiators were prepared by dissolving 3 wt% of St in 5 mL of DI water and 70–95 wt% of St in 5 mL of D.I water, respectively. The initiators were injected at a rate of 0.5 mL/s for 10 min using a syringe pump (NE300). Following the initiator injection, polymerization progressed for 5 h. The soap-free emulsion polymerization process was completed by rapidly decreasing the temperature to 30 °C at the end.

Then, PEI was added to PSMA solutions with varying concentrations of MA, based on the primary amine number. PEI has 569 free amino groups per mole, with 37.5, 38.1, and 24.1% of primary, secondary, and tertiary amines, respectively. MA and PEI were added in molar ratios of 1:0.02–0.16; PEI was injected into 10 mL of distilled water in varying proportions and dissolved for 30 min to prepare the PEI solution. The prepared PEI solution of 10 mL was added to 10 mL of PSMA, which was stirred at 150 rpm for 24 h to fabricate the PSMA/PEI core–shell nanoparticles. Subsequently, the solution was allowed to precipitate, and after removing the supernatant and separating the particles, centrifugation (1736R, 8000 rpm, 5 min) was performed thrice using ethanol to remove the unreacted PEI. The washed particles were dried in a vacuum oven at 30 °C for 12 h to obtain solid-state core–shell nanoparticles.

### 2.3. Chemical Analysis of PSMA and Core–Shell Nanoparticles

Cross-polarization, magic-angle spinning ^13^C nuclear magnetic resonance spectroscopic analysis (^13^C-CP/MAS NMR, Bruker, Billerica, MA, USA, Avance II, 500 MHz) was performed to ensure the completion of the PSMA and PSMA/PEI reactions. The rotor containing the sample was run using these specifications: 4 mm ZrO, 5 KHz spinning, 90° pulse, and 3.2 μs duration, with a contact time and repetition time of 0.02520 ms and 600 s, respectively. Attenuated total reflection–Fourier transform infrared spectroscopic analysis (ATR-FTIR, Italy) was performed using Nicolet 6700. Furthermore, elemental analysis (EA) was performed using an elemental analyzer (Flash 2000, Rivara, Italy) to measure the elemental composition of the sample.

### 2.4. Morphological Analysis of PSMA and Core–Shell Nanoparticles

Morphological analysis of the polymerized nanoparticles was performed via scanning electron microscopy (SEM, JEOL JSM6700, Tokyo, Japan); drops of polymerized emulsion nanoparticles were placed on a silicon wafer and dried at 25 °C for 10 h prior to the analysis. The core–shell structure of the nanoparticles was observed via transmission electron microscopy (TEM, JEM-F200,Tokyo, Japan). The sample was prepared by placing one or two drops dispersed in DI water of PSMA and the PSMA/PEI complex on carbon-coated copper grids and drying at 25 °C for 12 h. The atomic distribution of elements in the PSMA/PEI core–shell nanostructure was evaluated using energy-dispersive X-ray spectroscopy (EDS). The particle size distribution in the synthesized nanoparticles was analyzed using a Zetasizer nano system (Zetasizer nano ZS90, Malvern, UK), and the diluted MA sample was analyzed following further dilution to approximately 1 wt% using DI water.

## 3. Results and Discussion

### 3.1. Chemical Analysis of PSMA and Core–Shell Nanoparticles

The results of the solid-state ^13^C-NMR analysis for PSMA are shown in Figure S1. The peak corresponding to the methylene group owing to terminal cumene is observed at 28–49 and 117–128 ppm. A novel bimodal peak corresponding to the reaction between the amine and carboxyl groups is detected at 163–172 ppm for PSMA/PEI core–shell nanoparticles. The characteristic peaks of the carbonyl and carboxyl groups of MA were observed at 1859, 1778, and 1695 cm^−1^ in the FT-IR spectra. We also observed amide I and amide II peaks. The peak at 1650 cm^−1^ was due to amide C=O symmetric stretching. The amide N-H bending was observed at 1570 cm^−1^. The formation of the core–shell particles through the reaction with PEI is confirmed by the presence of amide peaks (1359, 1440, and 3202 cm^−1^) and reduced carbonyl peaks (1440 and 1359 cm^−1^). In addition, a decrease was observed in the CO stretching band at 1265 cm^−1^, which confirmed that a reaction occurred between the carboxyl groups of the open ring of MA and PEI, implying the successful binding of PEI to PSMA. These results are in agreement with the published reports [36].

The elemental compositions of PSMA and PSMA/PEI are listed in Table 1 and Table 2, respectively. The composition of PSMA compound carbon in St and oxygen and hydrogen in MA was measured. The variation in the elemental composition of the compound based on the addition of MA is listed in Table 1; for PS, the lack of MA prevented the detection of oxygen, whereas oxygen was detected in PSMA samples owing to the MA addition. The percentage of oxygen in PSMA increased with the wt% of MA, and at the highest wt% of 30, the atomic ratio of O was 26.83%, indicating a stable PSMA (92.6% conversion, Mw 262,000) polymerization.

The PSMA/PEI core–shell nanostructure was fabricated using PSMA as the core owing to its high oxygen proportion in the carbonyl group. PEI was added to the core in ratios to fabricate the core–shell nanoparticles as specified in Table 2. The EA of carbon in St, oxygen in MA, and nitrogen and hydrogen in PEI was conducted. The lack of PEI in PSMA prevents the detection of nitrogen, which was detected in all the other samples listed in Table 2 owing to the addition of PEI; the wt% of hydrogen was remarkably increased. The amount of oxygen decreased, but those of nitrogen and hydrogen increased for samples PSMA/PEI-1 to PSMA/PEI-4, which may be because of the intense reaction between the carboxyl groups of MA and amine groups, following the increase in the addition of PEI. However, for samples PSMA/PEI-5 to PSMA/PEI-8, the amounts of carbon, oxygen, nitrogen, and hydrogen did not change significantly. This might be because the reactive carboxyl and amine groups reached the maximum level in PSMA/PEI-5. In conclusion, PSMA with the highest proportion of oxygen was selected as the core, and for the resulting PSMA/PEI, PSMA/PEI-4 with the maximum level of reaction between the oxygen in MA and nitrogen in PEI was selected as the core–shell nanoparticle for further analysis.

### 3.2. Morphological Characteristics of PSMA Based on Various Factors

**Figure 1:**
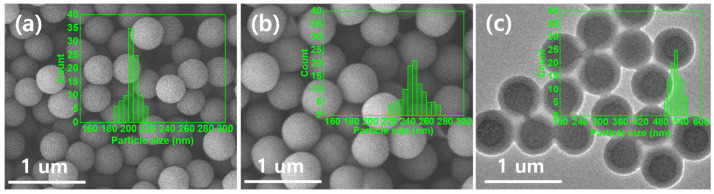
Scanning electron microscopy (SEM) images of PSMA nanoparticles prepared with different maleic anhydride (MA) ratios: (**a**) 5 and (**b**) 30 wt%. (**c**) Transmission electron microscopy (TEM) image of PSMA/PEI core–shell nanoparticles.

Figure 1 illustrates that the prepared PSMA and core–shell nanoparticles are spherical, uniform, and well dispersed. Furthermore, the SEM images show that the PSMA particle size tends to increase slightly as the MA ratio increases (Figure 1a,b). When the MA ratio was increased from 5 to 30 wt%, the average particle size distribution increased to approximately 45 nm. This was due to the surfactant effect of MA in the soap-free emulsion system. When PEI is added to the PSMA nanoparticles, the particle size increases from 220 nm to 522 nm up to a PEI:MA ratio of 0.08 (Figure 1c).

**Figure 2:**
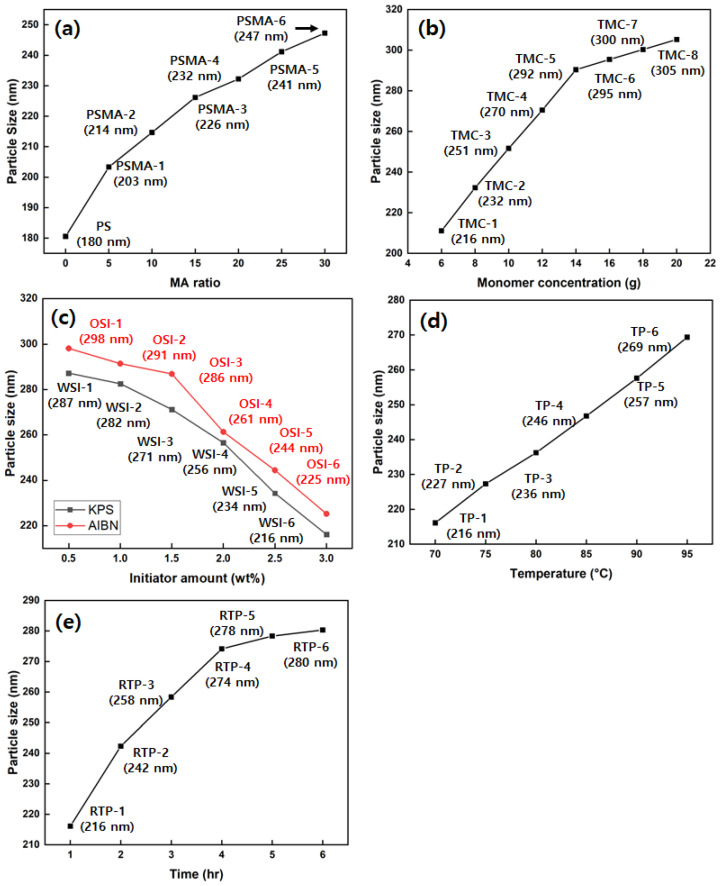
Particle size of PSMA plotted against the (**a**) monomer (MA) ratio, (**b**) monomer concentration, (**c**) initiator amount, (**d**) reaction temperature, and (**e**) reaction time.

**Figure 3:**
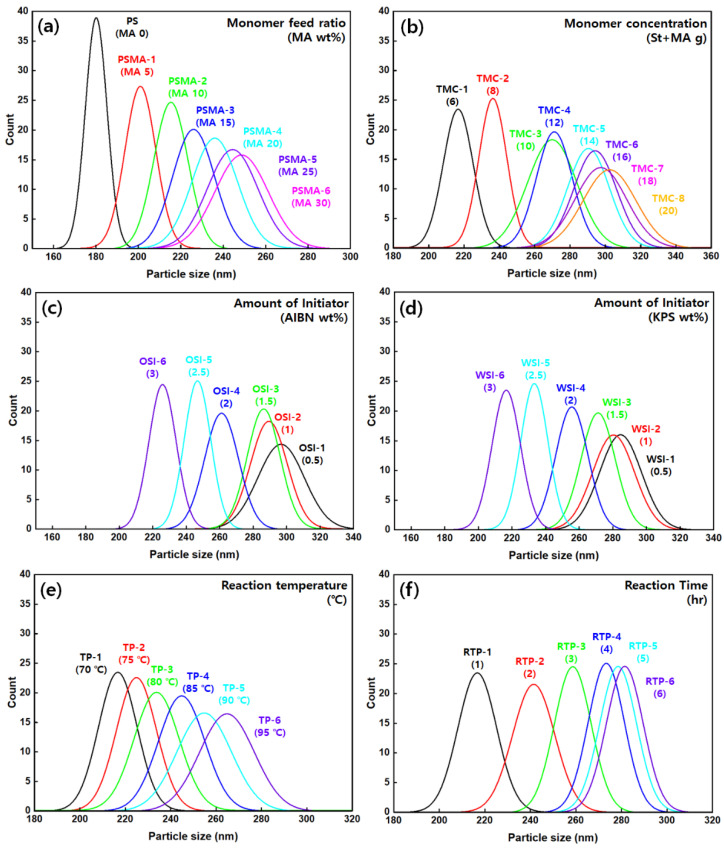
Particle size distribution of PSMA with respect to the (**a**) monomer (MA) ratio, (**b**) monomer concentration, (**c**) effect of oil-soluble initiator (AIBN), (**d**) effect of water-soluble initiator (KPS), (**e**) reaction temperature, and (**f**) reaction time.

To examine the variation in the morphology of PS and PSMA nanoparticles owing to the addition of St and MA, the nanoparticles were fabricated using various molar ratios, as presented in Table S1, and the particle size change is shown in Figure 2a, Figure 3a and Figure S2. The results indicated the formation of homogeneous particles. The smallest particle size was 180 nm for PS, and the particle size of PSMA increased substantially with the addition of MA. The largest particle size was observed for the PSMA sample having the highest proportion of MA.

As shown in Figure 2a, Figure 3a and Figure S2, the particle size and its distribution were the smallest and narrowest, respectively, for PS, in comparison with those of the other PSMA samples. This is because of the changes in the amount of the hydrophobic monomer St. During fabrication of PSMA using soap-free emulsion polymerization, St acts as a stabilizer; hence, an increase in St leads to a decrease in the particle size and, hence, narrowing of the particle size distribution. Figure 2b, Figure 3b and Figure S3 show the morphological changes owing to the differences in the concentrations of monomers. The monomers, St and MA were added in the molar ratio of 1:1 and stirred at 250 rpm for 6 h at 70 °C. The total amount of monomers was varied as 6, 8, 10, 12, 14, 16, 18, and 20 g/L. The increase in the particle size owing to the addition of monomers is shown in Figure 2b.

The particle size gradually increased with the amount of monomer, and as shown in Figure 3b, the distribution of particle size widened. This is due to the increased monomer concentration, which increased the number of reactants involved in the reaction. The time taken for complete monomer consumption and particle stabilization increased, resulting in a widened particle size distribution. Hence, TMC-1, with the smallest particle size and the narrowest distribution, was selected for analysis.

To examine the influence of the amount of initiator on the morphology of PSMA, oil-soluble AIBN and water-soluble KPS were added as 0.5, 1, 1.5, 2, 2.5, and 3% of the monomer ratio, as shown in Table S2. The particle size and distribution were obtained by adding the initiators to the TMC-1 sample. PSMA was synthesized by stirring at a speed of 250 rpm for 6 h at 70 °C. The observed morphological changes caused by the initiator are shown in Figure 2 and Figures S4 and S5. Increasing the amount of AIBN and KPS decreased the particle size.

Figure 2c, Figure 3c,d and Figures S4 and S5 show that irrespective of the initiator used, the particle size decreased, while the distribution narrowed as the amount of initiator increased. The particle size decreased because for an amount of dispersion media, a particular amount of monomers is dissolved to engage in the reaction, and when the amount of the initiator increases, the reaction occurs for the divided area of the monomers to meet the increased radicals.

Furthermore, the narrowed particle distribution may be attributed to the increase in particle stabilization. The increase in initiator concentration resulted in an increase in the generation of SO_4_ radicals and the initial number of particles; hence, the monomers were dissipated more rapidly than in low initiator concentrations. Thus, among the water-soluble KPS samples, WSI-6, which had the smallest particle size and narrowest distribution, was selected for analysis.

The morphological changes in the synthesized PSMA owing to the reaction conditions, such as temperature and reaction time, were analyzed. To examine the influence of reaction temperature on the PSMA morphology, the temperature was varied as shown in Table S3. TMC-1 was used to obtain the particle size and distribution for various monomer ratios and the added amount of St and MA. In addition, the stirring speed and reaction time were set to 250 rpm and 6 h, respectively, with the initiator WSI-6, for the synthesis of PSMA. The results are shown in Figure 3d and Figure S6.

Figure 2d and Figure 3e depict that the increase in reaction temperature from TP-1 (70 °C) to TP-6 (95 °C) increased the particle size and distribution. This is because of the reduced effect of the stabilizer with increased solubility but reduced solution viscosity at high reaction temperatures, which in turn raises the probability of collision among the primary particles, thereby increasing the particle size and distribution.

Furthermore, to examine the influence of reaction time on the morphology of PSMA, the reaction time was varied from 1 to 6 h, while the rest of the conditions were set as shown in Table S4 for the synthesis of the PSMA sample. The results are shown in Figure 2e and Figure S7. The increase in the reaction time increased the particle size, which was almost the same for samples prepared at a reaction time longer than 5 h.

Similarly, as shown in Figure 3f, the particle size increased with an increase in reaction time, while the particle distribution remained constant. Based on the mechanism of dispersion polymerization, oligomer radicals are formed in a homogeneous solution, and as the polymerized chain increases in length, the liquid phase starts to precipitate; hence, the primary nucleus acquires a strong tendency to assemble into a larger entity. However, as the reaction time increases, the agglomeration of the precipitated-polymer chain increases the number of nuclei and the particle size. A further increase in the number of polymerized particles results in the absorption of monomers and initiators into the interior of the particle, and as a result, the polymerization advances from the continuous phase toward the particle phase. Subsequently, when the particles have absorbed all the generated polymer chains, no new nuclei can be created; hence, the nucleation stage ends, and the particle size becomes constant.

### 3.3. Core–Shell Nanoparticle Size Distribution Based on the Feed Ratio of PEI

**Figure 4:**
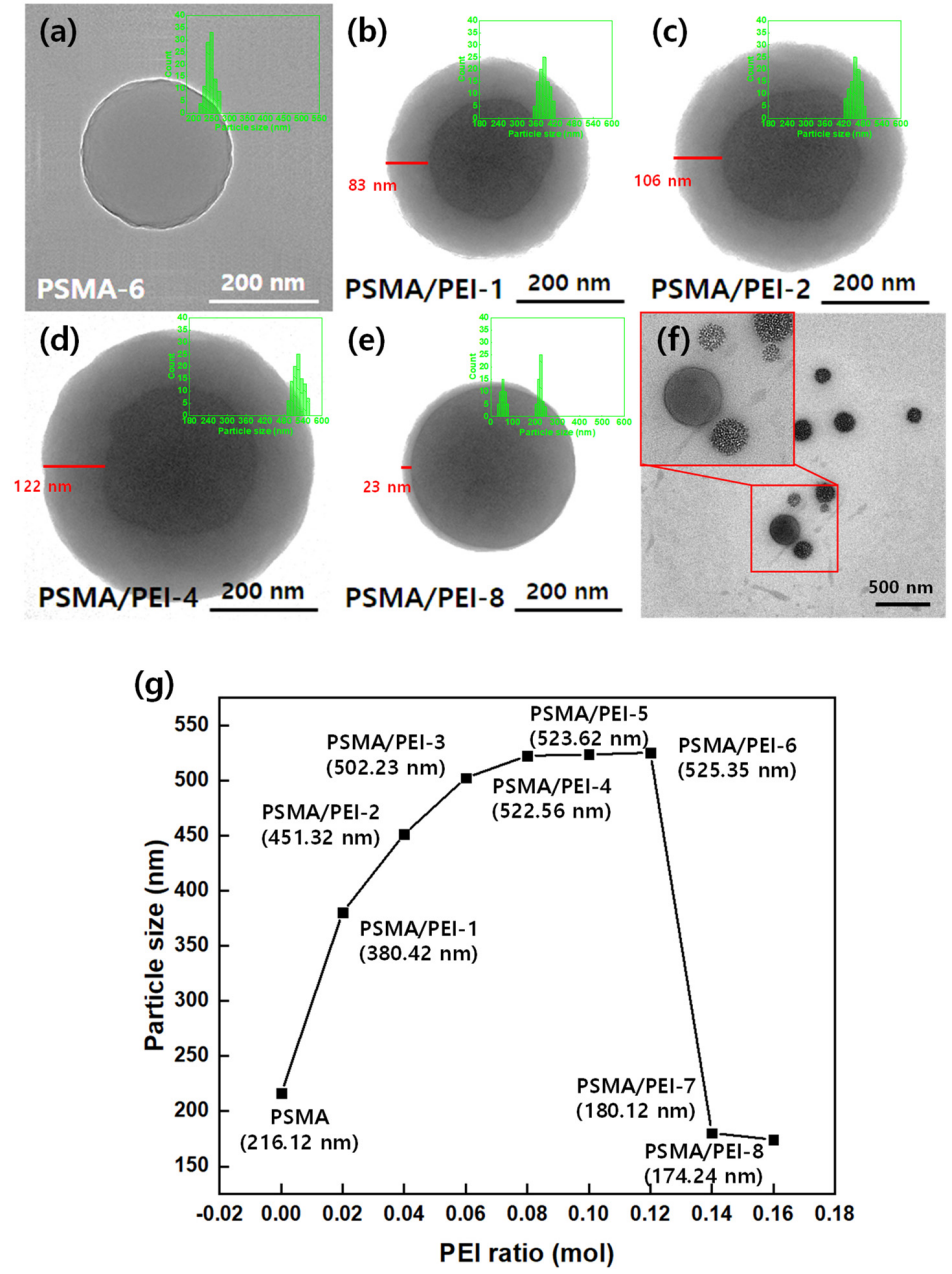
TEM images of PSMA/PEI core–shell nanoparticles with different shell thickness by the addition of PEI: (**a**) PSMA, (**b**) PSMA/PEI-1 (PEI = 0.02 mol), (**c**) PSMA/PEI-2 (PEI = 0.04 mol), (**d**) PSMA/PEI-4 (PEI = 0.08 mol), and (**e**) PSMA/PEI-8 (PEI = 0.16 mol); (**f**,**g**) dependence of particle size distribution on PEI amount in PSMA/PEI core–shell nanoparticles.

To investigate the morphology of the PSMA/PEI core–shell nanoparticles following the addition of PEI to PSMA, PSMA was selected as the core owing to its highest oxygen proportion in the carbonyl group. PEI was added to PSMA according to the molar ratios listed in Table 2. Changes in the PSMA/PEI core–shell particle size owing to the differences in the amount of PEI added were reported in a previous study [36]; however, the morphological changes induced by varying the feed ratios of PEI and particle dispersion were not mentioned. Thus, in this study, the changes in the particle size and distribution were analyzed based on PEI addition and interpreted as shown in Figure 4a–f.

In Figure 4g, the variation in particle size between the PSMA core without PEI addition and the PSMA/PEI-1 core–shell following the addition of PEI can be seen. In addition, the particle size increased for PSMA/PEI-1–PSMA/PEI-4, those of PSMA/PEI-5 and PSMA/PEI-6 exhibited resemblance, and those of PSMA/PEI-7 and PSMA/PEI-8 (174 nm) were found to have decreased.

The TEM images in Figure 4b–f also show that the shell thickness gradually increased from PSMA/PEI-1 to PSMA/PEI-4; for PSMA/PEI-5 and PSMA/PEI-6, the thicknesses were similar, and for PSMA/PEI-7 and PSMA/PEI-8, the shell thickness decreased. This variation in the thickness coincides with that of the particle size. The particle distribution was stable and narrow from PSMA/PEI-1 to PSMA/PEI-4, whereas it steadily increased from PSMA/PEI-5 until two peaks were detected from PSMA/PEI-7. This is due to the shell formation based on the reaction of the carboxyl groups in PEI and MA. In the case of PSMA without PEI feed, the lack of shell formation resulted in the smallest particle size, whereas for PSMA/PEI-1–PSMA/PEI-4, the PEI caused shell formation. Moreover, as the PEI feed ratio increased, the higher frequency of reaction resulted in an increased shell thickness and particle size. Furthermore, the increase in the shell thickness and particle size leveled off from PSMA/PEI-5 onwards because the reaction between the carboxyl group and PEI reached a maximum level at PSMA/PEI-4. The particle size began to decrease after PSMA/PEI-7, and the particle dispersion showed two peaks at 50 and 220 nm, attributed to the excessive addition of PEI. This resulted in the self-assembly of PEI exceeding the force of MA and PEI interaction for producing secondary particles. This caused the mean particle size to decrease and the particle dispersion to exhibit two peaks. Hence, for the fabrication of the PSMA/PEI core–shell particles, the PEI-to-MA ratio of 0.08 was the most efficient, which is in agreement with the findings of the EA.

## 4. Conclusions

We prepared PSMA/PEI core–shell nanoparticles for morphological analysis using the soap-free emulsion method. During the polymerization of PSMA, the addition of up to 30% MA resulted in a stable polymerization, and the atomic ratios of carbon, oxygen, and hydrogen were 72.92, 26.83, and 0.19%, respectively. During the fabrication of PSMA/PEI nanoparticles, the nitrogen content did not increase further once the reaction between the carboxyl and amine groups reached a maximum level. The number of reactants participating in the reaction increased depending on that of the initial monomer feed, with an increase in the particle size. Moreover, until the monomer consumption increased, the particle distribution widened. This is due to the increased monomer concentration, which increased the number of reactants involved in the reaction. The time taken for complete monomer consumption and particle stabilization increased, resulting in a widened particle size distribution. Varying the St content resulted in St acting as a stabilizer and reducing the particle size, and hence, the particle distribution narrowed. This is due to changes in the amount of the hydrophobic monomer St. This indicated that the feed ratio and amount of monomers changed the morphology. The increased amount of the initiator indicated that the reaction with radicals occurred for the divided area of the same amount of monomers; therefore, the particle size decreased, while the monomers were consumed more rapidly than at low initiator concentrations, irrespective of the type of initiator. Consequently, the particle stability increased, and the particle distribution narrowed. Furthermore, the morphological changes in PSMA owing to various reaction conditions, such as temperature, stirring speed, and reaction time, were analyzed. The results showed that as the temperature increased, the probability of collision among the primary particles increased, thus increasing the particle size and distribution. However, increasing the stirring speed increased the particle size up to a specific point, followed by a decrease. This is because stirring lowered the monomer solubility and interfered with the dispersion of RSO4- oligo radicals in the particle interior. The increased reaction time resulted in a larger particle size because the number of nuclei increased owing to the agglomeration of the polymer chain. The shell thickness and particle size were dependent on the PEI feed ratio in the fabrication of PSMA/PEI core–shell nanoparticles; the PEI-to-MA ratio of 0.08 was the most efficient. As such PSMA/PEI core–shell nanoparticles can be easily modified, this approach can be adapted to gas sensors, laying the foundation for future research in various areas, such as accident prevention in the chemical industry.

## Figures and Tables

**Table 1:** Elemental analysis (EA) of PSMA based on the addition of maleic anhydride (MA).

Sample Name	Ratio (wt%)		Atomic Ratio (%)		
	Copolymer of Styrene (St)	MA	C	O	H
PS	100	0	99.84	X	0.16
PSMA-1	95	5	96.69	3.06	0.25
PSMA-2	90	10	90.16	9.63	0.21
PSMA-3	85	15	86.76	13.01	0.23
PSMA-4	80	20	80.28	19.54	0.18
PSMA-5	75	25	75.47	24.31	0.22
PSMA-6	70	30	72.92	26.83	0.25

**Table 2:** EA of PSMA/PEI core–shell nanoparticles based on the addition of polyethylene imine (PEI).

Sample Name	Ratio (mol)	Atomic Ratio (%)			
	PEI:MA	C	N	O	H
PSMA	0:1	72.92	X	26.83	0.19
PSMA/PEI-1	0.02:1	72.44	7.43	11.15	8.98
PSMA/PEI-2	0.04:1	71.92	9.79	7.65	10.64
PSMA/PEI-3	0.06:1	72.58	10.54	5.76	11.12
PSMA/PEI-4	0.08:1	72.34	12.57	4.48	10.61
PSMA/PEI-5	0.10:1	71.57	13.01	4.54	10.88
PSMA/PEI-6	0.12:1	72.23	13.35	4.32	10.10
PSMA/PEI-7	0.14:1	72.06	6.55	12.21	9.18
PSMA/PEI-8	0.16:1	73.93	4.58	13.32	8.17

## Data Availability

The data presented in this study are available on request from the corresponding author.

## Supplementary Materials

The following are available online at https://www.mdpi.com/article/10.3390/nano11081958/s1, Figure S1: ^13^C-NMR spectra (500 MHz) of (a) PSMA and (b) PSMA/PEI; (c) FT-IR spectra of PSMA/PEI core–shell nanoparticles, Figure S2: Particle size distribution of PSMA with respect to the monomer feed ratio: (a) particle size distribution dependence on amount of MA in PSMA nanoparticles; (b) SEM images of PSMA nanoparticles prepared using different concentrations of MA, Figure S3: Particle size distribution of PSMA with respect to the monomer amount: (a) particle size distribution dependence on monomer amount in PSMA nanoparticles; (b) SEM images of PSMA nanoparticles prepared using different amounts of the monomer, Figure S4: Particle size distribution of PSMA based on amount of AIBN: (a) particle size distribution dependence on initiator type (AIBN) and amount in PSMA nanoparticles; (b–d) SEM images of PSMA nanoparticles prepared using different quantities of AIBN, Figure S5: Particle size distribution of PSMA based on amount of KPS used: (a) particle size distribution dependence on the amount of KPS in PSMA nanoparticles; (b–d) SEM images of PSMA nanoparticles prepared using different quantities of KPS, Figure S6: Particle size distribution of PSMA based on reaction temperature: (a) particle size distribution dependence of PSMA nanoparticles on the reaction temperature; (b) SEM images of PSMA nanoparticles prepared at different reaction temperatures, Figure S7: Particle size distribution of PSMA based on reaction time: (a) particle size distribution dependence of PSMA nanoparticles on the reaction time; (b) SEM images of PSMA nanoparticles prepared for different reaction times, Table S1: Samples prepared to analyze the variation in particle size and distribution of PSMA for various monomer concentrations, Table S2: Samples prepared to analyze the effect of the amount of initiator on particle size and distribution of PSMA, Table S3: Samples prepared to analyze the effect of varying reaction temperature on the particle size and distribution of PSMA, Table S4: Samples prepared to analyze the effect of reaction time on the particle size and distribution of PSMA.
