# Supplementary material for: Morphological Analysis of PSMA/PEI Core–Shell Nanoparticles Synthesized by Soap-Free Emulsion Polymerization
Source: Nanomaterials (Basel). 2021 Jul 29;11(8):1958. doi: 10.3390/nano11081958 (PMC8402240; doi:10.3390/nano11081958)
Supplement: Supplementary file 1 [file nanomaterials-11-01958-s001.zip › nanomaterials-1293142-supplementary.pdf]

nanomaterials

MDPI

Supplementary Materials

Morphological Analysis of PSMA/PEI Core–Shell Nanoparticles Synthesized by Soap-Free Emulsion Polymerization

Jae-Jung Park [1,†], Yongsoo Kim [2,†], Chanmin Lee [2], Donghyun Kim [1,2], Wonjun Choi [1,2], Hyukjun Kwon [1,2], Jung-Hyun Kim [1], Ki-Seob Hwang [2,*] and Jun-Young Lee [2,*]

[1] Department of Chemical and Biomolecular Engineering, Yonsei University Yonsei-ro 50, Seodaemun-gu, Seoul 120749, Korea; qkrwownd11@yonsei.ac.kr (J.-J.P.); ratian36@kitech.re.kr (D.K.); qwerty042@kitech.re.kr (W.C.); hyukjun@kitech.re.kr (H.K.); jaykim@yonsei.ac.kr (J.-H.K.)

[2] Research Institute of Sustainable Manufacturing System, Intelligent Sustainable Materials R&D Group, Korea Institute of Industrial Technology, 89 Yangdaegiro-gil, Ipjang-myeon, Seobuk-gu, Cheonan-si, Chungcheongnam-do 31056, Korea; bohemian4215@kitech.re.kr (Y.K.); clee@kitech.re.kr (C.L.)

* Correspondence: ks_hwang@kitech.re.kr (K.-S.H.); jaylee@kitech.re.kr (J.-Y.L.); Tel.: +82-41-5898-424 (J.-Y.L.)

† These authors contributed equally to this work.

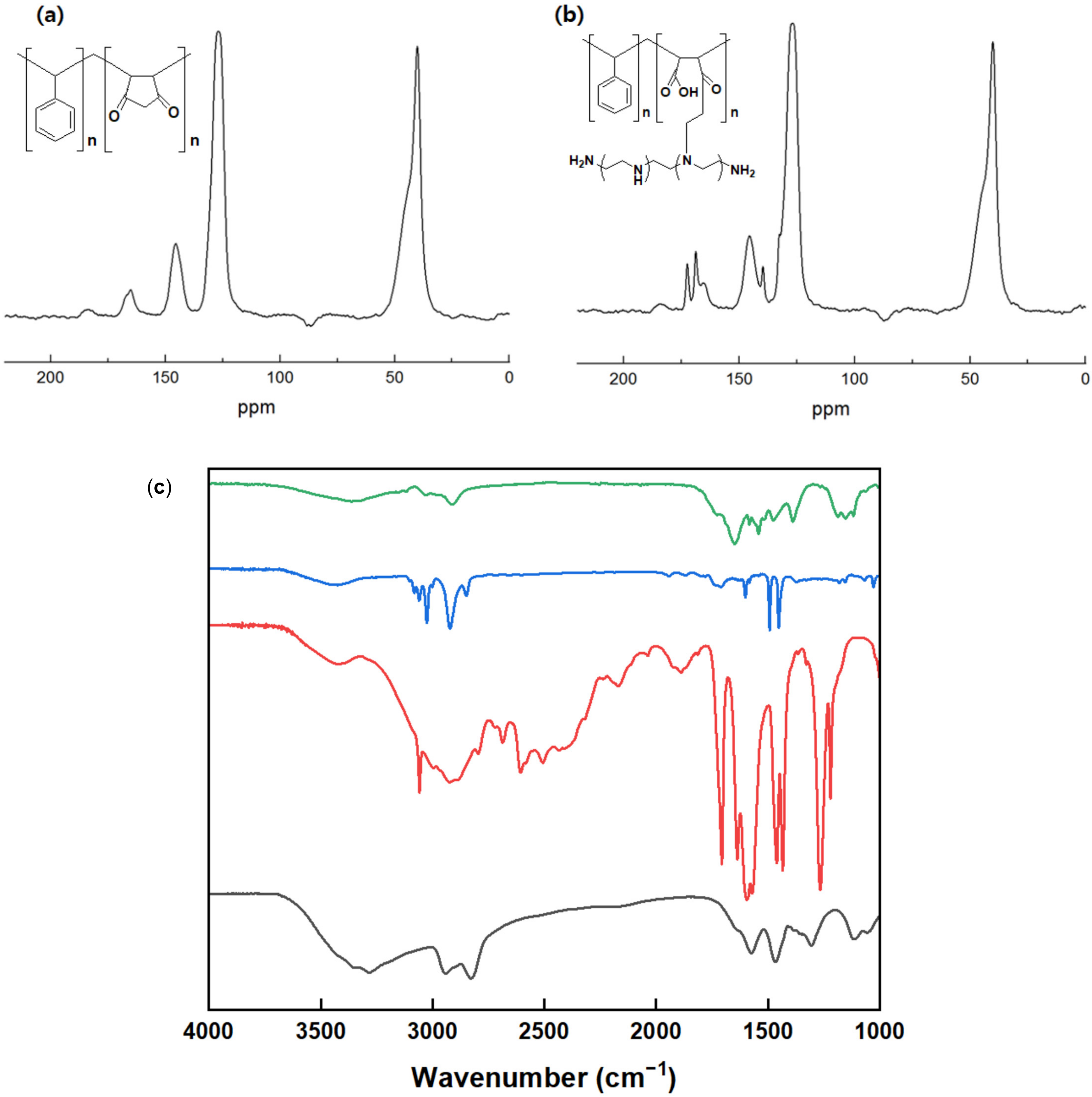

Figure S1. ^{13}C-NMR spectra (500 MHz) of (**a**) PSMA and (**b**) PSMA/PEI; (**c**) FT-IR spectra of PSMA/PEI core–shell nanoparticles.

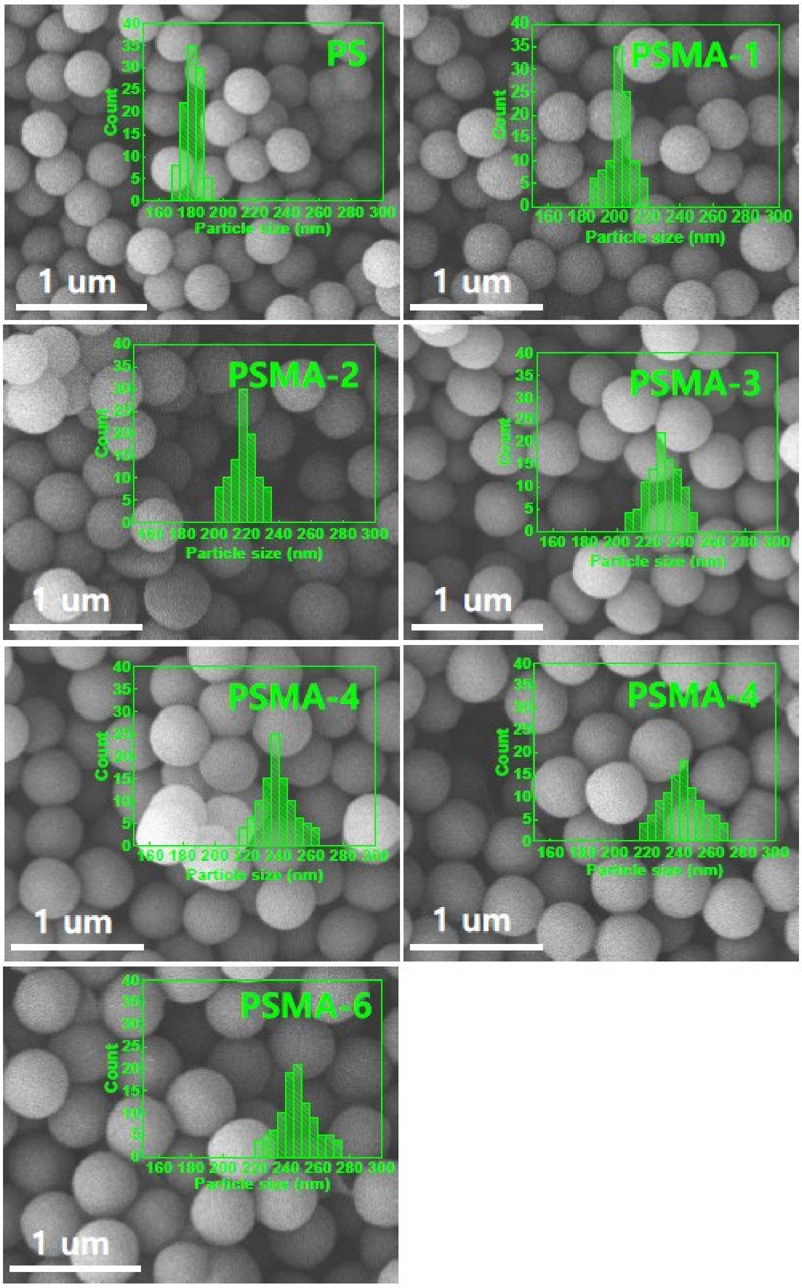

Figure S2. Particle size distribution of PSMA with respect to the monomer feed ratio: (**a**) particle size distribution dependence on amount of MA in PSMA nanoparticles; (**b**) SEM images of PSMA nanoparticles prepared using different concentrations of MA.

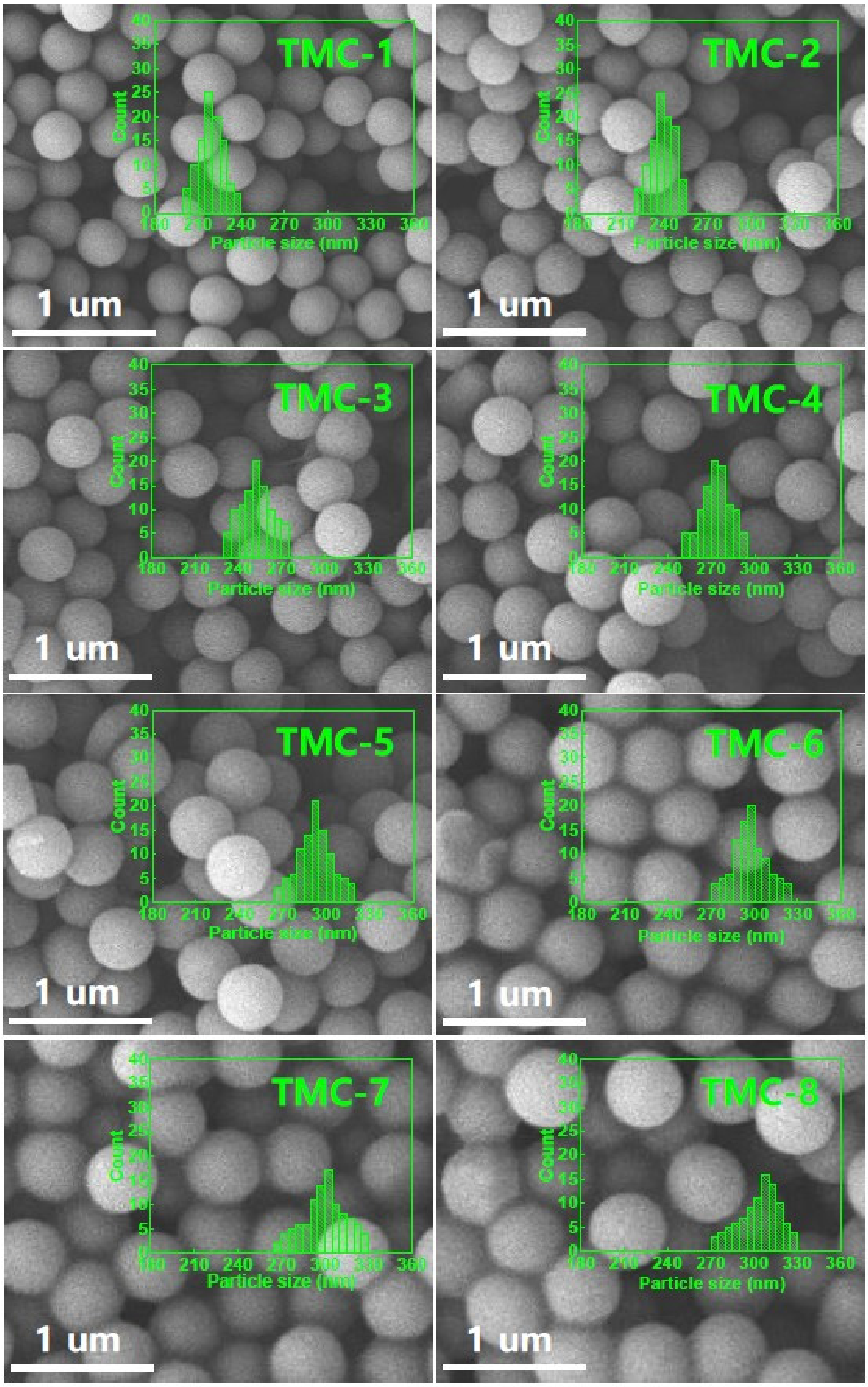

Figure S3. Particle size distribution of PSMA with respect to the monomer amount: (**a**) particle size distribution dependence on monomer amount in PSMA nanoparticles; (**b**) SEM images of PSMA nanoparticles prepared using different amounts of the monomer.

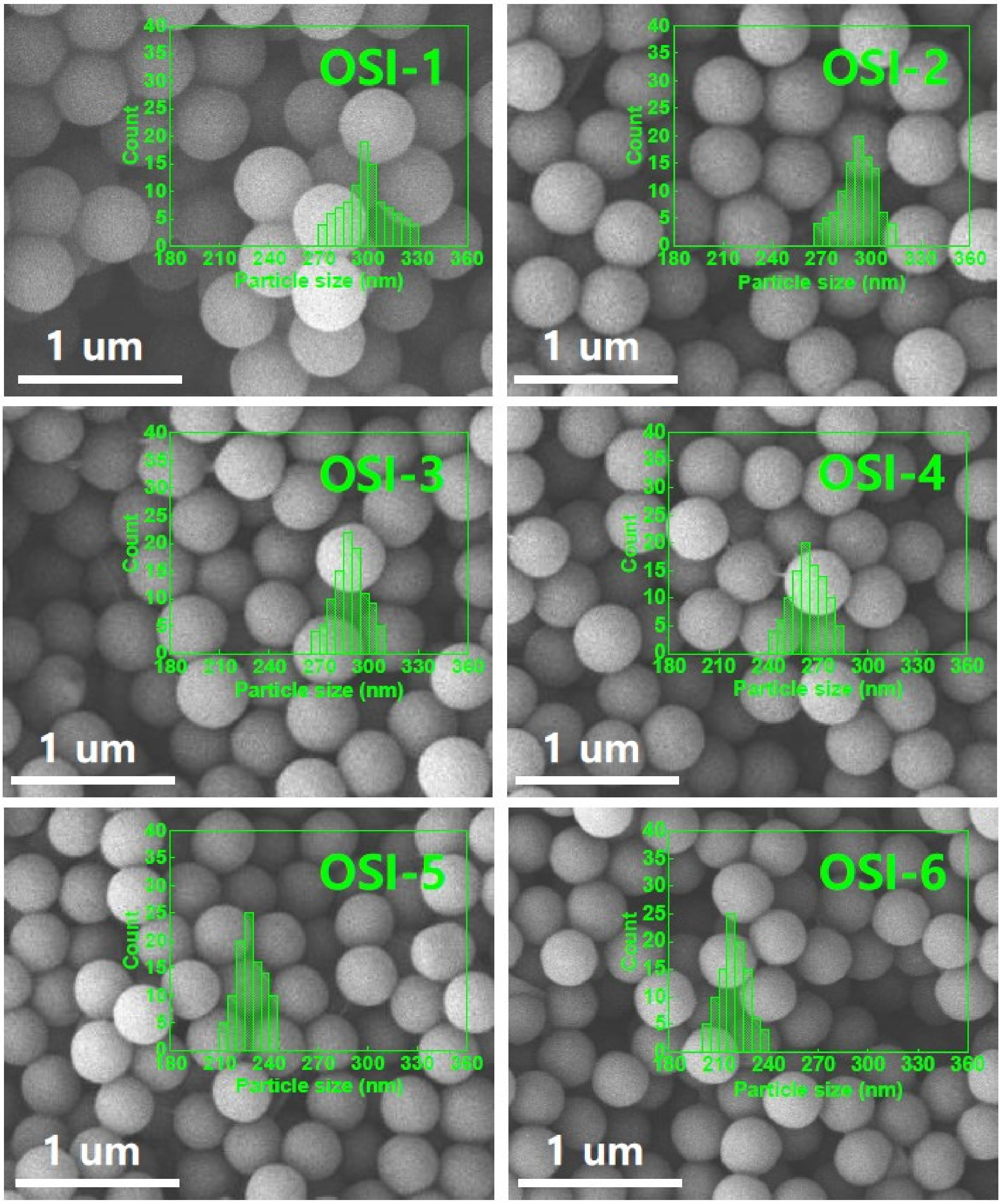

Figure S4. Particle size distribution of PSMA based on amount of AIBN: (**a**) particle size distribution dependence on initiator type (AIBN) and amount in PSMA nanoparticles; (**b**–**d**) SEM images of PSMA nanoparticles prepared using different quantities of AIBN.

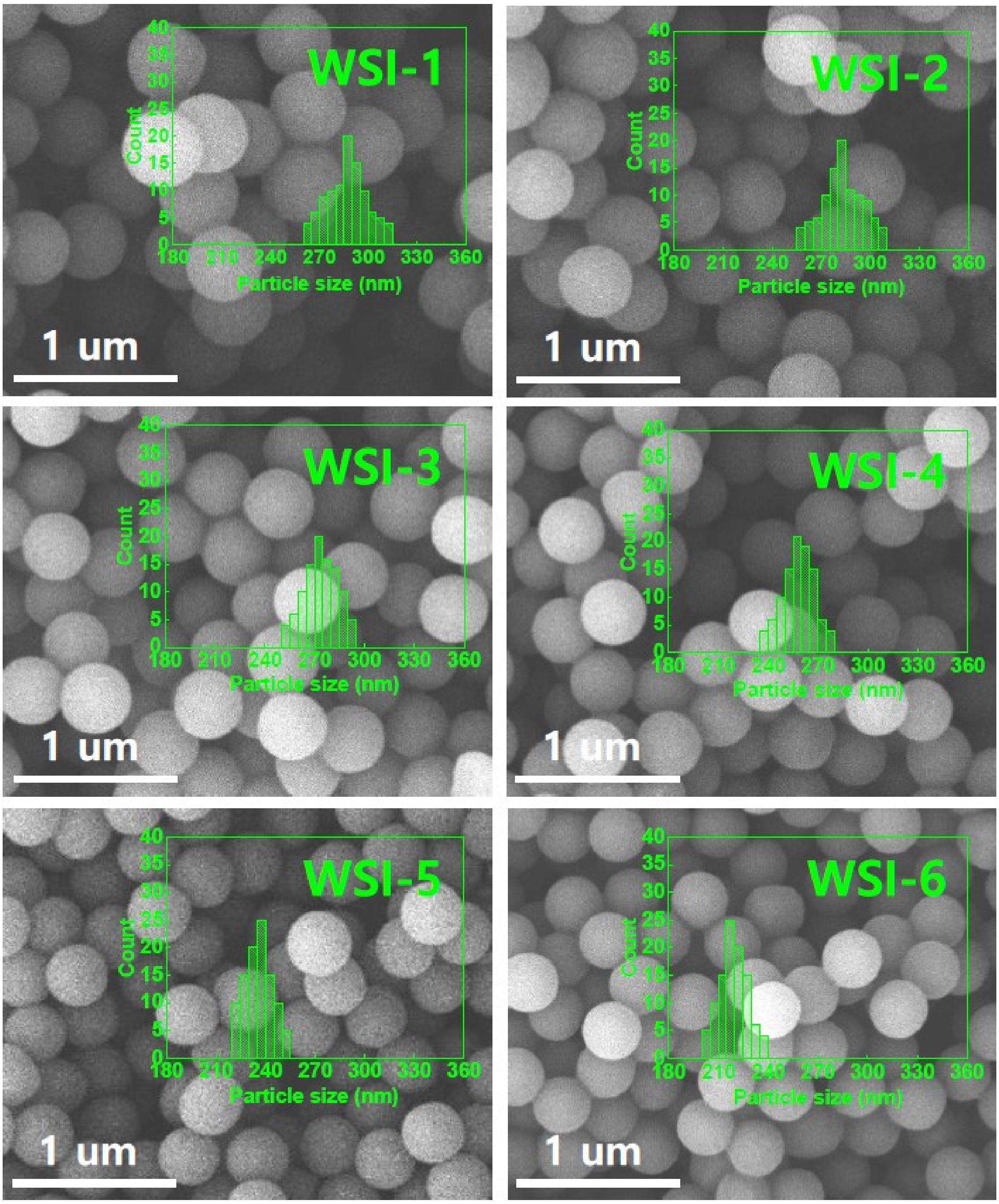

Figure S5. Particle size distribution of PSMA based on amount of KPS used: (**a**) particle size distribution dependence on the amount of KPS in PSMA nanoparticles; (**b**–**d**) SEM images of PSMA nanoparticles prepared using different quantities of KPS.

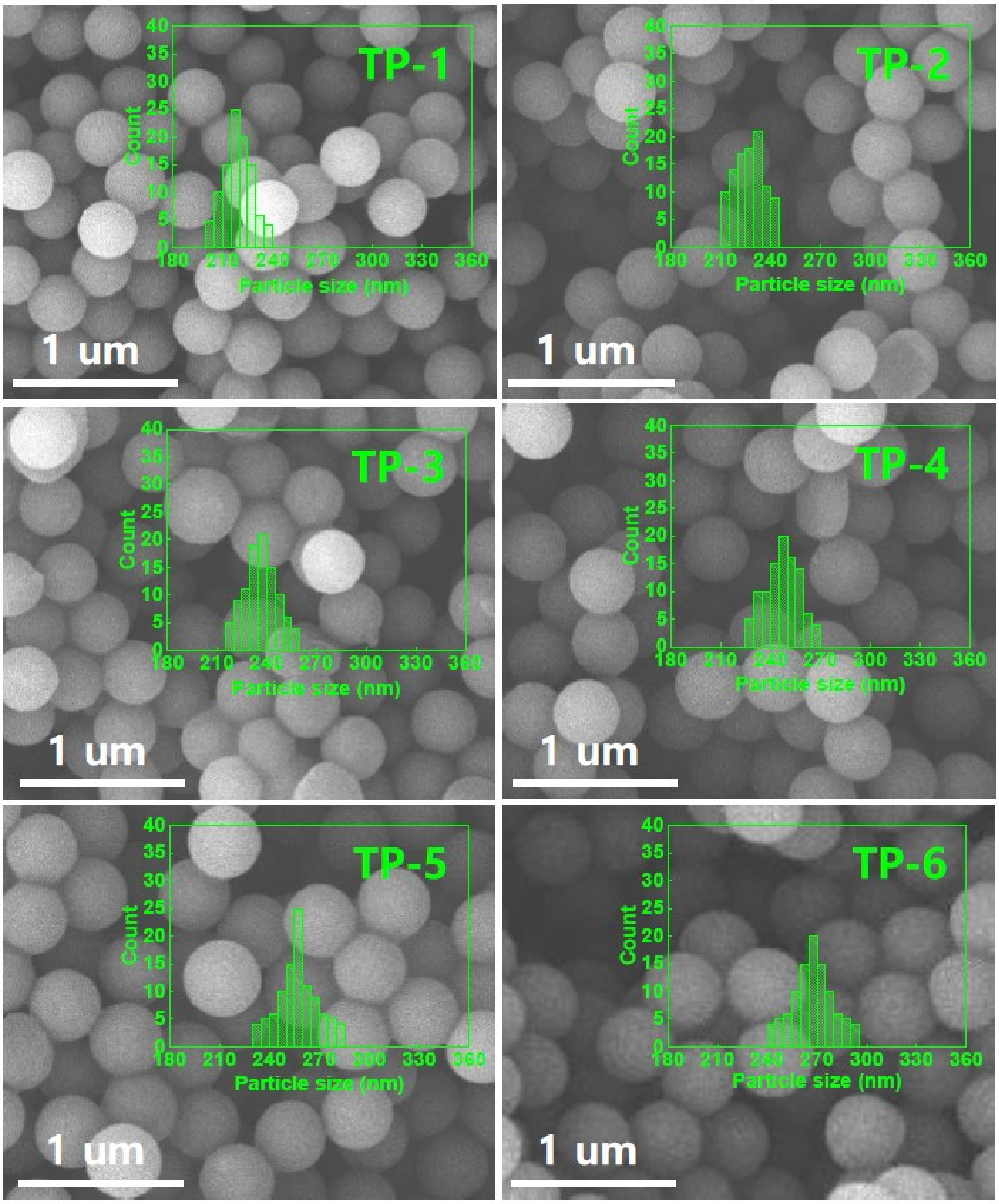

Figure S6. Particle size distribution of PSMA based on reaction temperature (°C): (**a**) particle size distribution dependence of PSMA nanoparticles on the reaction temperature (°C); (**b**) SEM images of PSMA nanoparticles prepared at different reaction temperatures (°C).

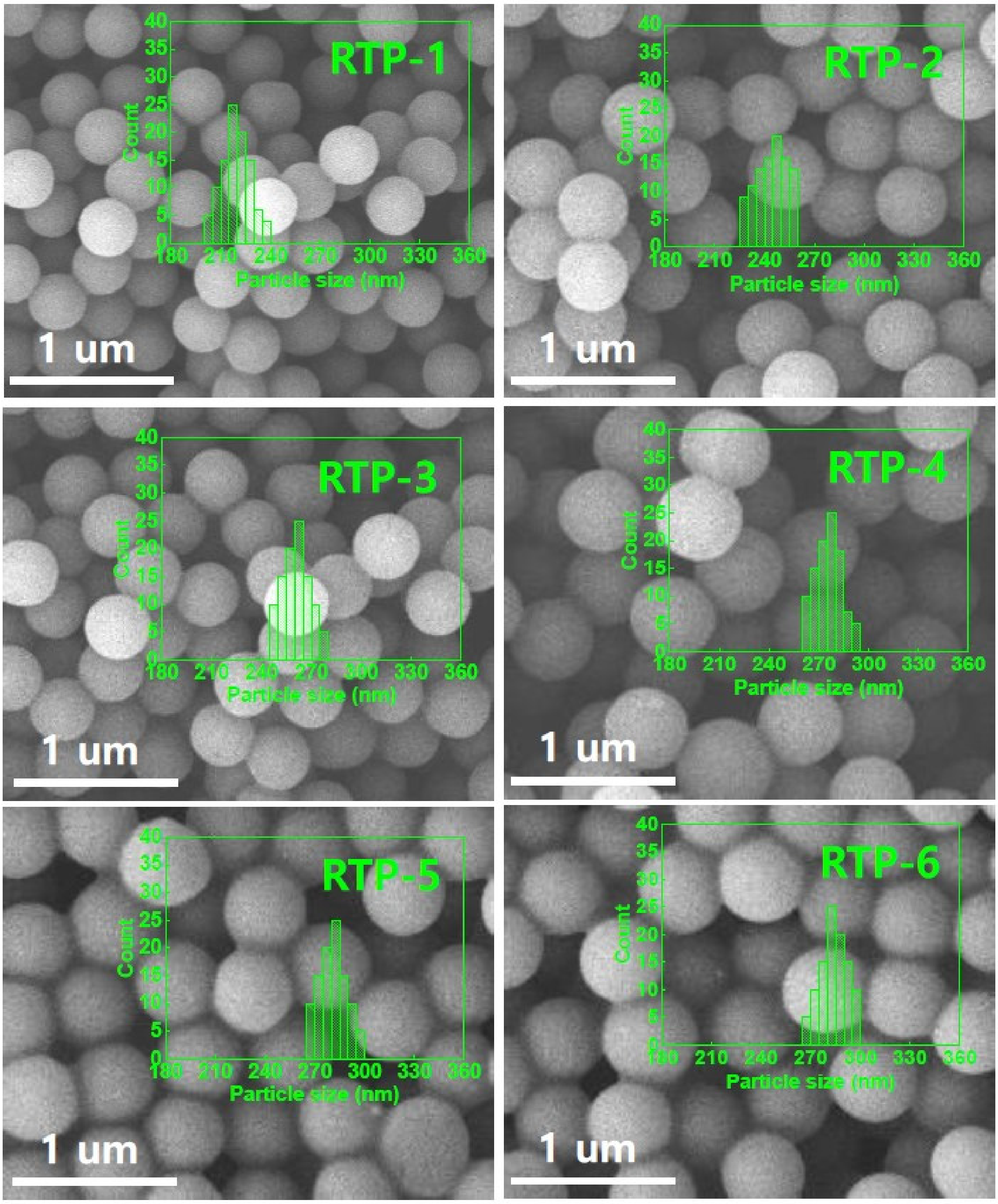

Figure S7. Particle size distribution of PSMA based on reaction time: (**a**) particle size distribution dependence of PSMA nanoparticles on the reaction time; (**b**) SEM images of PSMA nanoparticles prepared for different reaction times.

Table S1. Samples prepared to analyze the variation in particle size and distribution of PSMA for various monomer concentrations.

Sample Name	Monomer ratio (mol)		Total monomer concentration
	ST	MA	
TMC-1	1	1	6
TMC-2	1	1	8
TMC-3	1	1	10
TMC-4	1	1	12
TMC-5	1	1	14
TMC-6	1	1	16
TMC-7	1	1	18
TMC-8	1	1	20

Table S2. Samples prepared to analyze the effect of the amount of initiator on particle size and distribution of PSMA.

Sample Name	Initiator		Monomer ratio (mol)	
	type	Amount (wt%)	St	MA
OSL-1	AIBN	0.5	1	1
OSL-2		1		
OSL-3		1.5		
OSL-4		2		
OSL-5		2.5		
OSL-6		3		
WSI-1	KPS	0.5		
WSI-2		1		
WSI-3		1.5		
WSI-4		2		
WSI-5		2.5		
WSI-6		3		

Table S3. Samples prepared to analyze the effect of varying reaction temperature on the particle size and distribution of PSMA.

Sample name	Temperature (°C)	Reaction time (h)	Stir speed (rpm)	Monomer	Initiator
TP-1	70	6	250	TMC-1	WSI-6
TP-2	75				
TP-3	80				
TP-4	85				
TP-5	90				
TP-6	95				

Table S4. Samples prepared to analyze the effect of reaction time on the particle size and distribution of PSMA.

Sample name	Reaction time (h)	Stir speed (rpm)	Temperature (°C)	Monomer	Initiator
RTP-1	1	250	70	TMC-1	WSI-6
RTP-2	2				
RTP-3	3				
RTP-4	4				
RTP-5	5				
RTP-6	6				
